# Causes, outcomes and diagnosis of acute breathlessness hospital admissions in Malawi: protocol for a multicentre prospective cohort study

**DOI:** 10.12688/wellcomeopenres.21041.1

**Published:** 2024-04-17

**Authors:** Stephen A. Spencer, Florence Malowa, David McCarty, Elizabeth Joekes, Jacob Phulusa, Beatrice Chinoko, Sylvester Kaimba, Lucy Keyala, Peter Mandala, Mercy Mkandawire, Matthew Mlongoti, Bright Mnesa, Albert Mukatipa, Rhona Mijumbi, Mulinda Nyirenda, Hendry R. Sawe, Marc Henrion, Daniel X. Augustine, David Oxborough, Eve Worrall, Felix Limbani, Paul Dark, Stephen B. Gordon, Jamie Rylance, Ben Morton, Collaborators

**Affiliations:** 1 Department of Clinical Sciences, Liverpool School of Tropical Medicine, Liverpool, UK; 2 Malawi-Liverpool-Wellcome Trust Clinical Research Programme, Blantyre, Southern Region, Malawi; 3 Queen Elizabeth Central Hospital, Blantyre, Southern Region, Malawi; 4 The Kamuzu University of Health Sciences, Blantyre, Southern Region, Malawi; 5 Emergency Medicine Department, Muhimbili University of Health and Allied Sciences, Dar es Salaam, Tanzania; 6 Royal United Hospitals Bath NHS Foundation Trust, Bath, England, UK; 7 Liverpool Centre for Cardiovascular Sciences, Liverpool John Moores University, Liverpool, England, UK; 8 Humanitarian and Conflict Response Institute, The University of Manchester, Manchester, England, UK

**Keywords:** Breathlessness, respiratory distress, sub-Saharan Africa, hospital care, emergency care, low-resource settings

## Abstract

**Background:**

Hospital admission due to breathlessness carries a significant burden to patients and healthcare systems, particularly impacting people in low-income countries. Prompt appropriate treatment is vital to improve outcomes, but this relies on accurate diagnostic tests which are of limited availability in resource-constrained settings. We will provide an accurate description of acute breathlessness presentations in a multicentre prospective cohort study in Malawi, a low resource setting in Southern Africa, and explore approaches to strengthen diagnostic capacity.

**Objectives:**

Primary objective: Delineate between causes of breathlessness among adults admitted to hospital in Malawi and report disease prevalence. Secondary objectives **:** Determine patient outcomes, including mortality and hospital readmission 90 days after admission; determine the diagnostic accuracy of biomarkers to differentiate between heart failure and respiratory infections (such as pneumonia) including brain natriuretic peptides, procalcitonin and C-reactive protein.

**Methods:**

This is a prospective longitudinal cohort study of adults (≥18 years) admitted to hospital with breathlessness across two hospitals: 1) Queen Elizabeth Central Hospital, Blantyre, Malawi; 2) Chiradzulu District Hospital, Chiradzulu, Malawi. Patients will be consecutively recruited within 24 hours of emergency presentation and followed-up until 90 days from hospital admission. We will conduct enhanced diagnostic tests with robust quality assurance and quality control to determine estimates of disease pathology. Diagnostic case definitions were selected following a systematic literature search.

**Discussion:**

This study will provide detailed epidemiological description of adult hospital admissions due to breathlessness in low-income settings, which is currently poorly understood. We will delineate between causes using established case definitions and conduct nested diagnostic evaluation. The results have the potential to facilitate development of interventions targeted to strengthen diagnostic capacity, enable prompt and appropriate treatment, and ultimately improve both patient care and outcomes.

## Plain language summary

Background: People admitted to hospital with symptoms of breathlessness are often severely ill and need quick, accurate assessment to facilitate timely initiation of appropriate treatments. In low resource settings, such as Malawi, limited access to diagnostic equipment impedes patient assessment. Failure to identify and treat the underlying diagnosis may lead to preventable death.

Aims: This cohort study aims to delineate between common, treatable causes of breathlessness among adult patients admitted to hospital in Malawi and measure survival. We will also evaluate the performance of blood markers to diagnose and differentiate between conditions. The results will help us develop context-appropriate diagnostic and treatment algorithms based on resources available in the health system

Methods in brief: We will recruit adult patients who present to hospital with breathlessness in a central national referral hospital (Queen Elizabeth Central Hospital, Blantyre), and a district hospital (Chiradzulu District Hospital, Chiradzulu). We will conduct enhanced diagnostic tests to determine causes of breathlessness against internationally accepted diagnostic guidelines. Patients will be followed up throughout their hospital admission and after discharge, until 90 days.

Interpretation: This study aligns with World Health Assembly resolutions on ‘Strengthening diagnostics capacity’ and on ‘Integrated emergency, critical and operative care for universal health coverage and protection from health emergencies’. The results of this study will have the potential to facilitate development of interventions targeted to strengthen diagnostic capacity, enable prompt and appropriate treatment, and ultimately improve care and outcomes for acutely unwell patients.

## Introduction

Globally, admission to hospital with breathlessness is common and carries significant burden to both patients and healthcare systems, particularly in low resource settings ^1, 2^. Understanding the underlying causes of breathlessness is critical to ensure prompt initiation of appropriate treatment to improve patient outcomes. However, an estimated 47% of the global population have limited or no access to diagnostics, which disproportionately impacts those living in low- and middle-income countries ^3, 4^. This study aims to examine the causes of acute breathlessness presentations in Malawi, and to identify context-appropriate clinical factors that may improve diagnostic accuracy.

Studies investigating breathless-related hospital admissions in Africa have focused on paediatric populations, with epidemiology in adult hospital admissions poorly understood ^5, 6^. The burden amongst adults is high; hypoxaemic (low blood oxygen) presentations account for 10% (14/144) of medical admissions in Malawi ^7^. Mortality is also high; 42% (90/214) patients with hypoxaemia in a Rwanda cohort died in-hospital ^8^. Breathless adults are often critically ill, and require delivery of prompt emergency care to prevent death ^6^. However, limited resources, infrastructural limitations and a high prevalence of communicable and non-communicable diseases contribute to the complexity of diagnosing and treating patients with acute breathlessness ^9^.

We conducted a systematic literature search to identify up-to-date diagnostic guidelines for conditions that lead to breathlessness, based on parameters available in resource-constrained settings. This process has informed the development of the diagnostic methodology within our protocol.

This multicentre prospective observational study will systematically examine causes of breathlessness and outcomes among patients admitted to hospitals in Malawi. We will also evaluate diagnostic performance of biomarkers for heart failure and acute respiratory infections (such as pneumonia) which have potential to improve initial clinical assessment and management for patients with acute breathlessness. These results are intended to inform the development of targeted interventions, enhance context-appropriate diagnostic capabilities, and improve the management and patient outcomes for those suffering from breathlessness in low-resource settings.

### Breathlessness definition

Dyspnoea, the symptom of breathlessness, is not always associated with hypoxaemia. We will adopt a broad definition of breathlessness that includes symptoms (dyspnoea) and physiological parameters. This will allow us to capture medical conditions that are not perceived as breathlessness but have objective signs of respiratory distress and include patients who are tachypnoeic (defined as a respiratory rate [RR] ≥ 25); and/or are hypoxaemic (SpO2 < 94%); and/or require supplemental oxygen therapy. These physiological threshold levels are based on criteria from UK National Early Warning Score (NEWS) 2 ^10^. This will ensure that our study is relevant to the maximum number of patients who present to hospital “short of breath”.

### Objectives

The primary objective is to understand the causes of breathlessness as defined above and report disease prevalence. We will utilise diagnostic techniques to differentiate between causes of breathlessness, including heart failure, respiratory infections (including pneumonia and tuberculosis), anaemia, pulmonary embolism, pulmonary arterial hypertension, asthma, chronic obstructive pulmonary disease, myocardial infarction, pleural effusion and pneumothorax. We will focus on identifying and delineating common treatable conditions that can lead to breathlessness, and hypothesise that the largest of cases will likely be heart failure and pneumonia ^1, 11^.

Secondary and exploratory objectives are presented in Table 1. Briefly, secondary objectives include assessment of patient outcomes (90 day mortality, readmission rate and hospital length of stay); assessment of diagnostic accuracy of brain-natriuretic peptide for heart failure and both C-reactive protein and procalcitonin for respiratory infections (such as pneumonia). Exploratory objectives include derivation and internal validation of a clinical prediction model to differentiate between common causes of breathlessness (with a focus on heart failure and respiratory infections such as pneumonia); cost-evaluation on the tests required to diagnose heart and respiratory infections (pneumonia).

**Table 1.** Objectives and outcome measures.

Objectives	Outcome measures
***Primary objective for observational clinical study*** • Determine the prevalence of conditions that cause breathlessness within study population	• Presence of disease, determined by internationally recognised case definitions **
***Secondary objectives for observational clinical study*** • Determine survival 90 days after index presentation * • Determine re-admission rate 90 days after index admission * • Hospital length of stay	• Survival determined if the patient is alive 90 days after the date of index hospital admission • Hospital readmission defined as an unplanned acute admission with an overnight stay • Duration of stay in hospital for the acute admission, from time and date of admission until time and date of discharge
***Secondary objectives for diagnostic accuracy study*** • Assess the diagnostic accuracy of natriuretic peptides (BNP and NT-proBNP) for heart failure • Assess the diagnostic accuracy of C-reactive protein (CRP) and procalcitonin (PCT) for respiratory infections (e.g. pneumonia)	• Sensitivity, specificity, area under the receiver operator curve (AUROC) ^§^ • Sensitivity, specificity, area under the receiver operator curve (AUROC) ^§^
***Exploratory outcomes*** • Derive and internally validate a clinical prediction model for heart failure and respiratory infections (e.g. pneumonia) diagnoses based on parameters available in a typical district hospital • Cost evaluation for each diagnostic test for heart failure and respiratory infections (e.g. pneumonia) and their associated diagnostic accuracy	• Performance indicators including measures of discrimination (e.g. c-statistic); measures of calibration (e.g. calibration plots); measures of clinically relevant performance (e.g. positive predictive value, negative predictive value) • Determine the cost implications of diagnostic tests, and compare costs with the test diagnostic accuracy

*We may also assess outcomes at 12 months (telephone appointment). **Methodological guidance will be followed in instances of missing or incomplete data (for example, by consensus medical diagnosis) ^13^.

## Protocol

### Study design

A multicentre prospective cohort study of medical admissions to hospital with breathlessness in Malawi. This study is nested within a programme of work titled *Multimorbidity-associated emergency hospital admissions: a screen and link strategy to improve outcomes for high-risk patients in sub-Saharan Africa* (MultiLink study; ( https://multilinknihr.com). The MultiLink cohort is a prospective observational study that aims to identify multimorbidity among patients admitted to hospital in Malawi and Tanzania. This will be achieved by systematically screening acutely admitted patients for multimorbidity using enhanced point of care diagnostic tests at the point of entry to hospital. The protocol for the MultiLink cohort study has been published elsewhere ^12^.

Recruitment for the current study will begin in parallel across sites in September 2022. We have provided details of study schedule including follow-up in Table 2. 

**Table 2.** Study procedures and sampling schedule.

Study Visit	A	B	C	D	E	F	G
Day post admission	0	2	5	7	Discharge	30	90
Deferred consent	x						
Consent (Verbal)	x	x	x	x	x		x
Consent (Written) ^§^	x	x	x				
Vital Signs	x	x	x	x	x		x
Medical History and outcome assessment	x					x	x
POC Brain Natriuretic Peptide (BNP), 2x POC Troponin (cTnI) ^†^							
Electrocardiogram (ECG)	x						
Serum: laboratory CRP, PCT, NT-proBNP; serum save (5ml)	x						x
Microbiology: Blood culture, sputum (Xpert Tb), nasopharyngeal swab (molecular respiratory panel)	x						
Imaging: CXR, echocardiogram and lung ultrasound	x						
Spirometry							x
Screen for AEs	x	x	x	x	x		x
HIV POC	x						x
Where HIV: viral load, urine LAM *	x						
Blood glucose POC	x						x
HbA1c POC	x						x
Creatinine POC	x						x
Urinary dipstick	x						x
EQ5D questionnaire	x					x	x

CXR: chest x-ray; BP: Blood pressure; POC: Point of care blood test; CRP: C-reactive protein; PCT: procalcitonin; BNP: brain natriuretic peptide; NT-proBNP: N-terminal pro-brain natriuretic peptide; AE: adverse effects; HIV: human immunodeficiency virus; urine LAM: urinary lipoarabinomannan; HbA1c: glycated haemoglobin. Additional contact with the participants may be made up to 12 months by telephone after hospital admission to check the longer-term outcomes. Visit E (hospital discharge) visit data will be collected at any point before or after day 2, 5 and 7 follow up visits. Visit F will be a telephone follow-up. Visit G will be an in-person outpatient follow-up clinic. There will be flexibility of ±10 working days for this follow up visit to take place. This study will provide the following tests which are not routinely available in the study hospitals: BNP, NT-proBNP, troponin, CRP, procalcitonin, spirometry. The study will also provide the following tests which are not routinely available in the district hospital health system: formal echocardiography and lung ultrasound; nasopharyngeal swab (for molecular analysis); blood culture testing. ^**†**^ 2 x serial POC troponin (cTnI) tests at baseline and 6–24 hours later. Blood and urine samples will be stored at -80°C for further testing. * Samples for urinary LAM will be collected and processed as part of routine clinical service to screen for opportunistic infection in line with WHO guidance at admission. ^§ ^Written consent will be taken at the earliest possible opportunity, either directly from the patient or from a proxy (if patient lacks capacity).

### Study setting

Patients will be recruited from two hospitals in Malawi: from a central/urban hospital (Queen Elizabeth Central Hospital (QECH), Blantyre), and a district /rural hospital (Chiradzulu District Hospital, Chiradzulu. QECH is a government (public) tertiary referral hospital in the southern region of Malawi, with a 1350-bed capacity. Due to the absence of secondary health centres in Blantyre, QECH also acts as a secondary level service. The hospital provides medical care for patients presenting directly from the community, those referred from local primary health centres or from secondary health centres in the Southern Malawi region (including from Chiradzulu District Hospital). Population projections from the Malawi National Statistical Office estimated the Blantyre city population in 2023 to be 871,776 and southern region population to be 7,912,347 ^14^. Chiradzulu District Hospital is a government secondary care hospital of 300 beds in the Southern Malawi region. Patients are admitted to Chiradzulu district hospital directly from the community or via referral from primary health centres. The Chiradzulu district population in 2023 is estimated to be 389,928 ^14^.

### Participants, schedule and timelines

Enrolment will take place at the point of hospital admission, with eligible participants consecutively recruited within 24 hours of presentation. Recruitment will be stratified across the two sites. Consent procedures have been previously described ^12^. Recruitment will be conducted by an experienced clinical research team at the Malawi-Liverpool-Wellcome (MLW) Programme following training in study standard operating procedures (SOPs).

### Eligibility criteria

Inclusion (meets all of):

1. Adult patients (≥18 years)
2. Decision to admit to hospital
3. Acute medical problem
4. Usual residence in the study catchment area (Blantyre district or Chiradzulu district)
5. Contactable by telephone after discharge (either directly or through a carer)
6. Presentation with one of the following symptoms or signs:
  a. Shortness of breath
  b. Respiratory rate (RR) ≥ 25/minute
  c. SpO _2_ <94%
  d. Supplemental oxygen therapy

Exclusion (meets any of):

1. Pregnancy (justification: other local research is specifically addressing this)
2. Planned (elective) medical admission
3. Admission for primary trauma, obstetric or gynaecological condition
4. Primary abdominal/surgical cause of breathlessness (e.g. peritonitis)
5. Detainees or prisoners
6. Patient or carer declines consent to take part

### Study outcome conditions

We will systematically screen patients presenting with breathlessness for common possible diagnoses (listed in Table 3).

**Table 3.** Diagnostic criteria and case definitions.

Condition	Diagnostic modalities	Case definitions	Sub-classification criteria
INFECTION			
Acute respiratory infection (ARI)	• History	National Institute for Health and Care Excellence (NICE; UK) ^15^ and European Respiratory Society (ERS) ^16^: • An acute illness (present for 21 days or less) affecting the respiratory tract with symptoms such as cough, sore throat, fever, sputum production, breathlessness, wheeze or chest discomfort or pain, and no alternative explanation.	Aetiology will be classified according to the pathogen: 1. ARI, aetiology not defined 2. ARI with confirmed pathogen: i. ARI with bacterial pathogen (excluding Mycobacterial Tuberculosis [MTB]) ii. ARI with viral pathogen iii. ARI with MTB
Severe Acute Respiratory Infection (SARI)	• History • Vital signs	WHO SARI definition ^17^: An acute respiratory illness with each of: • History of or measured fever (≥38 °C) • Cough • Onset within last 10 days • Requires hospitalisation	Aetiology will be classified according to the pathogen: 1. SARI, aetiology not defined 2. SARI with confirmed pathogen: i. SARI with bacterial pathogen (excluding MTB) ii. SARI with viral pathogen iii. SARI with MTB
Pneumonia	• History • Examination • Vital signs • Medications • CXR • Lung ultrasound • Blood culture • Molecular viral respiratory panel	Pneumonia diagnostic criteria will be based on the Infectious Diseases Society of America/American Thoracic Society (ATS) Consensus Guidelines ^18^: • “Clinical features (e.g. cough, fever, sputum production, and pleuritic chest pain) supported by a demonstrable infiltrate by chest radiograph or other imaging technique, with or without supporting microbiological data, is required for the diagnosis of pneumonia” Note: this definition also covers the definition of community-acquired pneumonia in guidelines from NICE ^15^ and ERS ^16^.	Aetiology of pneumonia will be classified according to the pathogen: 1. Pneumonia, aetiology not defined 2. Pneumonia with confirmed pathogen: i. Pneumonia with bacterial pathogen (excluding MTB) ii. Pneumonia with viral pathogen iii. Pneumonia with MTB
Tuberculosis (TB)	• History • Examination • Clinical diagnoses • Vital signs • TB treatment • CXR • Urine LAM • Xpert TB/RIF	Tuberculosis will be defined as: 1) Primary outcome. Microbiologically confirmed MTB If any of the following are positive ^19^: • Xpert MTB/RIF • Urine LAM 2) Secondary outcome. In the absence of any positive microbiological tests for MTB ^20^: • Clinical diagnosis of TB in the presence of either compatible clinical illness or radiological disease • And/or decision to start TB treatment by the responsible clinical team.	Tuberculosis sub-classification ^19^: 1. Pulmonary TB:positive Xpert MTB/RIF with absent or negative urine LAM. 2. Disseminated TB: positive urine LAM
CARDIAC			
Heart failure	• History • Examination • Vital signs • Medications • CXR • Lung ultrasound • Echocardiography	Heart failure diagnostic criteria will be based on the Universal Definition of Heart failure ^21^ * • Symptoms and/or signs of heart failure (see supplementary file 1 ^22^) • Evidence of a structural and/or functional cardiac abnormality, including at least one of: ○ EF <50% ○ abnormal cardiac chamber enlargement ○ E/E′ >15 ○ Moderate/severe ventricular hypertrophy ○ Moderate/severe valvular stenosis or regurgitation. • Objective evidence of cardiogenic or systemic congestion with available imaging: ○ CXR ○ Lung ultrasound ○ Elevated filling pressures by echocardiography. Note: natriuretic peptides, as one of the index tests of the study will not form part of the definition for the diagnostic accuracy study.	Heart failure will be sub-classified based on ejection fraction, systolic vs diastolic function; structural abnormality (morphology), staging and functional impact. **1) Ejection fraction ^21^:** • Heart failure with reduced ejection fraction (HFrEF): LVEF ≤40% • Heart failure with mildly-reduced ejection fraction (HFmrEF): LVEF 41-49% • Heart failure with preserved ejection fraction (HFpEF): LVEF ≥ 50% **2) Structural abnormality** (see supplementary file 1 ^22^) **3) Heart failure stage** ^21^: Stage A (at risk of heart failure); Stage B (pre-heart failure); Stage C (heart failure); Stage D (advanced heart failure) **4) Functional classification:** New York Heart Association functional classification (NYHA classes I-IV) ^21^. We will also identify patients with newly diagnosed heart failure, pre-existing chronic heart failure and those with ‘worsening of chronic heart failure’ (WHF). WHF will be defined in line with ESC criteria among patients with pre-existing heart failure with worsening of signs or symptoms of heart failure and require treatment intensification ^23^.
Myocardial infarction (MI)	• Symptoms • ECG • cTnI • Echo	Myocardial infarction will be defined in line with the Fourth Universal Definition of Myocardial Infarction ^24^ and will include criteria for the following (see sub-classification column for details): • Acute myocardial infarction ○ Type 1 MI ○ Type 2 MI ○ Type 3 MI • Prior MI Note: neither cardiac surgery nor coronary procedures (e.g. percutaneous coronary intervention) are available in Malawi and we do not expect to identify patients with cardiac/coronary procedural associated MI (Type 4 or 5) Definition of myocardial injury: a raised cTnI level with at least one value above the 99th percentile upper reference limit (URL) ^24^.	**1) Type 1 MI.** MI caused by atherosclerotic coronary artery disease. This will be diagnosed by: • Evidence of a rise and/or fall of cTnI values (>20% variation) with at least one value above the 99 ^th^ percentile. • and at least 1 of the following: ○ Symptoms of myocardial ischemia (see supplementary file 1) ○ Ischemic ECG changes (see supplementary file 1) ○ Pathological Q waves (see supplementary file 1) ○ Imaging (echocardiographic) evidence of loss of viable myocardium or regional wall motion abnormality in a pattern consistent with an ischemic aetiology. Note: identification of coronary thrombus by angiography or autopsy is not available in this setting and will not form part of our definition. **2) Type 2 MI:** MI caused by a mismatch between oxygen supply and demand, that is not due to atherosclerotic disease. Meets criteria for Type 1 MI, alongside evidence of an imbalance between oxygen supply and demand (e.g. evidence of at least one of: severe anaemia, respiratory failure, tachyarrhythmia, severe bradyarrhythmia, severe hypertension ± left ventricular hypertrophy, clinical shock/hypotension). **3) Type 3 MI:** Patients who have died following symptoms of MI with evidence of ECG changes of either MI or ventricular fibrillation, but have died before troponin levels taken. **4) Prior myocardial infarction.** Any one of the following: • Abnormal Q waves (with or without symptoms in the absence of non-ischemic causes). • Pathoanatomical findings of a prior MI. This will be detected as a regional wall motion abnormality via echocardiography. • Note nuclear medical techniques are not available in this setting and we will not be able to detect imaging evidence of loss of viable myocardium.
OBSTRUCTIVE LUNG DISEASES			
Obstructive ventilatory impairment	• Spirometry	We will follow ERS/ATS guidelines to define obstructive ventilatory impairment ^25^: • FEV1 / FVC < 5 ^th^ percentile (z-score < -1.645)	Obstructive lung impairment will be sub-classified by severity ^25^: • Normal: FEV1 z-score > −1.645 • Mild impairment: FEV1 z-score -1.65 to -2.5 • Moderate impairment: FEV1 z-score < -2.51 to -4 • Severe impairment: FEV1 z-score < -4.1
Chronic obstructive pulmonary disease (COPD)	• History • Spirometry	COPD will be defined following ERS/ATS criteria ^25, 26^: • FEV1 / FVC < 5 ^th^ percentile (z-score < -1.645) • No evidence of reversibility (a change in FEV1 of >10% is considered a significant bronchodilator response) ^25^ • Symptoms including chronic dyspnoea, chronic cough or sputum production	COPD will be sub-classified by severity ^25^: • Normal: FEV1 z-score > −1.645 • Mild impairment: FEV1 z-score -1.65 to -2.5 • Moderate impairment: FEV1 z-score < -2.51 to -4 • Severe impairment: FEV1 z-score < -4.1 The modified Medical Research Council (mMRC) dyspnoea scale will be used as an assessment of patient reported severity, in line with guidance ^27^.
Asthma	• History • ISAAC / GAN core asthma screening questionnaire • Medications • Clinical diagnoses • Spirometry	**1) Primary outcome:** Severe asthma exacerbation based on the ATS/ERS official definition for clinical trials ^28^: • “A hospitalisation because of asthma, requiring systemic corticosteroids” **2) Secondary outcomes:** i. Presence of asthma symptoms from International Study of Asthma and Allergies in Childhood (ISAAC) and Global Asthma Network (GAN) questionnaire ^29^. ii. Asthma diagnosis confirmed by reversible airflow obstruction defined as >10% change relative to an individual’s predicted FEV1 and FVC ^30^.	The following definitions will follow criteria from the ISAAC/ and GAN core screening questions ^29^: • “Current wheezing illness” will be defined as a “yes” response to the question: “Have you had wheezing or whistling in the chest in the past 12 months”? • “Severe wheezing” will be defined as current wheeze and any one of ^31^: ○ > 4 attacks of wheeze in the past 12 months ○ >1 night per week sleep disturbance from wheeze ○ wheeze affecting speech. • “Previous diagnosis of asthma” will be defined as a “yes” response to the question: “Have you ever had a diagnosis of asthma”? We will assess patient reported impact and severity using the mMRC dyspnoea scale ^32^. Note: all patients hospitalised due to an asthma exacerbation will be defined as having ‘uncontrolled asthma’, per ERS/ATS criteria ^33^.
HAEMATOLOGICAL / VASCULAR			
Anaemia	• POC haemoglobin (Hb)	WHO haemoglobin thresholds for the diagnosis of anaemia ^34^ • Anaemia in non-pregnant women (age ≥15 years): <120g/l • Anaemia in males (age ≥15 years): <130 g/l	Anaemia will be sub-classified by severity based on WHO thresholds ^34^. Anaemia in non-pregnant women (age ≥15 years): < 120g/l • Mild: 110-110 • Moderate: 80-109 • Severe: <80 Anaemia in males (age ≥15 years): < 130 g/l • Mild: 110-129 • Moderate: 80-109 • Severe: <80 Haemoglobin levels will be adjusted for altitude and smoking status per WHO guidelines ^34^
Pulmonary embolism (PE)	• Clinical notes • Echocardiography	We will define pulmonary embolism as: • CT proven PE (limited availability and not a routine study procedure) • Clinically suspected PE and treated with anticoagulants • Echocardiographic diagnoses In the absence of computed tomography (CT), echocardiography will be used to diagnose PE. • Confirmed PE (ERS/ESC guidelines ^35^): visualised thrombi in the right heart or pulmonary artery (or main branches of the pulmonary artery) will confirm the diagnosis of PE. • Suspected PE (ERS/ESC and BTS guidelines ^35, 37^): ○ High risk patient with haemodynamic instability (systolic BP < 90 mmHg, a drop of > 40 mmHg, or need for vasopressors to maintain systolic BP ≥90 mmHg) ^35^. ○ Echocardiographic findings of RV pressure overload (RV strain): RV dilation (basal RV/LV ratio >1.0); and/or McConnell’s sign (reduced contractility of the RV free wall and normal / hyperkinetic RV apical contractility); and/or D-sign (flattening/deviation of the interventricular septum towards the LV). ^35, 38^ ○ Absence of other clear causes of RV pressure overload. Note: the utility of echocardiographic findings of RV pressure overload to support diagnosis (and emergency treatment) of PE among haemodynamically unstable patients are endorsed by guidelines from the BTS ^29^, ERS ^35^, PERT Consortium ^39^ and JCS ^40^.	Sub-classification will be based on diagnostic modality and by American Heart Association (AHA)/ESC criteria ^35, 36^: 1) Massive PE (or high risk PE): Hypotension, defined as systolic BP <90 mm Hg, a drop of >40 mmHg, or need for vasopressors to maintain SBP ≥90 mmHg. 2) Submassive PE (or intermediate risk PE): echocardiographic signs of RV pressure overload (RV strain) without hypotension, or RV injury detected by increase in cardiac biomarkers (troponin, BNP, or NT-proBNP). 3) Low risk PE: PE that does not fit criteria for massive or submassive PE.
Pulmonary hypertension (PH)	• Echocardiography	We will follow established reference protocols from the BSE on the assessment of PH ^41^.	PH will be graded as low, intermediate or high probability in line with BSE criteria ^41^. PH will be classified as: • Post-capillary PH (PH due to left heart disease) • Pre-capillary PH (PH due to pulmonary arterial hypertension; PH due to lung disease; chronic thromboembolic pulmonary hypertension [CTEPH]; unclear or multifactorial causes of PH). Note: classification will be based on echocardiographic criteria; ^41^ right heart catheterisation to confirm diagnosis and subtype is not available in this setting.
PLEURAL DISEASE			
Pleural effusion / empyema	• CXR • Lung ultrasound	Evidence of pleural effusion on CXR or lung ultrasound. Pleural effusion will be identified sonographically as an anechoic or hypoechoic fluid collection in the pleural space ^42^.	Pleural effusion will be sub-classified by: 1) Maximum depth of effusion: • CXR criteria: Small (1 intercostal space [ICS]; moderate (2-3 ICS); large ≥4 ICS) • Lung ultrasound criteria: depth of effusion measured in cm perpendicular from the parietal pleura. 2) Simple vs complex effusion (assessed sonographically; see supplementary file 1 ^22^)
Pneumothorax	• CXR • Lung ultrasound	Evidence of pneumothorax on CXR or lung ultrasound. Pneumothorax will be identified on chest radiographs as a visible visceral pleural line with air between it and the chest wall and absent pulmonary vessels ^43^. Pneumothorax will be identified sonographically where each of the following are fulfilled (in line with international consensus guidelines) ^44^: • Absence of lung sliding • Absence of B-lines • Either presence of a lung point or absence of a lung pulse.	Pneumothorax will be sub-classified as radiographic of pneumothorax or of tension pneumothorax (see supplementary file 1 ^22^).

ARI: acute respiratory infection; ATS: American Thoracic Society; BNP: brain natriuretic peptide; BSE: British Society of Echocardiography; COPD: chronic obstructive pulmonary disease; CT: computed tomography; cTnI: cardiac troponin I; CXR: chest x-ray; E: mitral valve pulse wave peak early diastolic wave velocity; e’: mitral valve tissue doppler peak early diastolic wave velocity; ECG: electrocardiogram; EF: ejection fraction; ERS: European Respiratory Society; ESC: European Society of Cardiology; FEV1: forced expiratory volume in 1 second; FVC: forced vital capacity; Hb: haemoglobin; NICE: National Institute for Health and Care Excellence; HFmrEF: heart failure with mildly-reduced ejection fraction; HFpEF: heart failure with preserved ejection fraction; HFrEF: heart failure with reduced ejection fraction; ICS: intercostal space; ISAAC / GAN: International Study of Asthma and Allergies in Childhood / Global Asthma Network; JCS: Japanese Circulation Society; LV: left ventricle; LVEF: left ventricular ejection fraction; mMRC: modified Medical Research Council dyspnoea scale; MI: myocardial infarction; MTB: mycobacterial tuberculosis; NYHA: New York Heart Association; PE: pulmonary embolism; PERT: Pulmonary Embolism Response Team; PH: pulmonary arterial hypertension; RIF: rifampicin; RV: right ventricle; SARI: severe acute respiratory infection; TB: tuberculosis; urine LAM: urine lipoarabinomannan; URL: upper reference limit; WHO: World Health Organization. Note: information on diagnostic test methodology are available in supplementary file 1 ^22^, where the processes for acquisition, interpretation, quality assurance and quality control are described. * Some definitions have been adapted either due to i) resource availability or ii) for methodological reasons. The rational for these adaptations are as follows: a) Heart failure. The universal definition for heart failure includes the use of natriuretic peptides to corroborate a diagnosis of heart failure. One of our study aims is to assess the diagnostic accuracy and validate the use of natriuretic peptides in the southern African context; BNP will therefore not form part of the definition for this study. b) Acute myocardial infarction. Cardiac imaging (e.g. for assessment of wall motion abnormality) will be restricted to echocardiography alone due to resource availability. Angiography is not available in this setting and will not form part of our definition

We will focus on identifying and delineating common treatable conditions, which have been identified from existing literature ^45– 48^. For example, heart failure is a common treatable cause of admission to hospital in sub-Saharan Africa, which can be difficult to diagnose and differentiate from pneumonia, due to a lack of available diagnostic tools (e.g. echocardiography, radiology (such as chest x-ray [CXR]) and microbiology). Differentiating between heart failure and pneumonia is therefore an important focus for this study. Case definitions for all study outcome conditions are presented in Table 3, alongside criteria for sub-classification. As part of exploratory analyses we will also evaluate the prevalence of acute respiratory distress syndrome (ARDS) using criteria modified for low-resource settings in the New Global Definition of ARDS ^49, 50^.

### Diagnostic techniques

**Figure 1.**
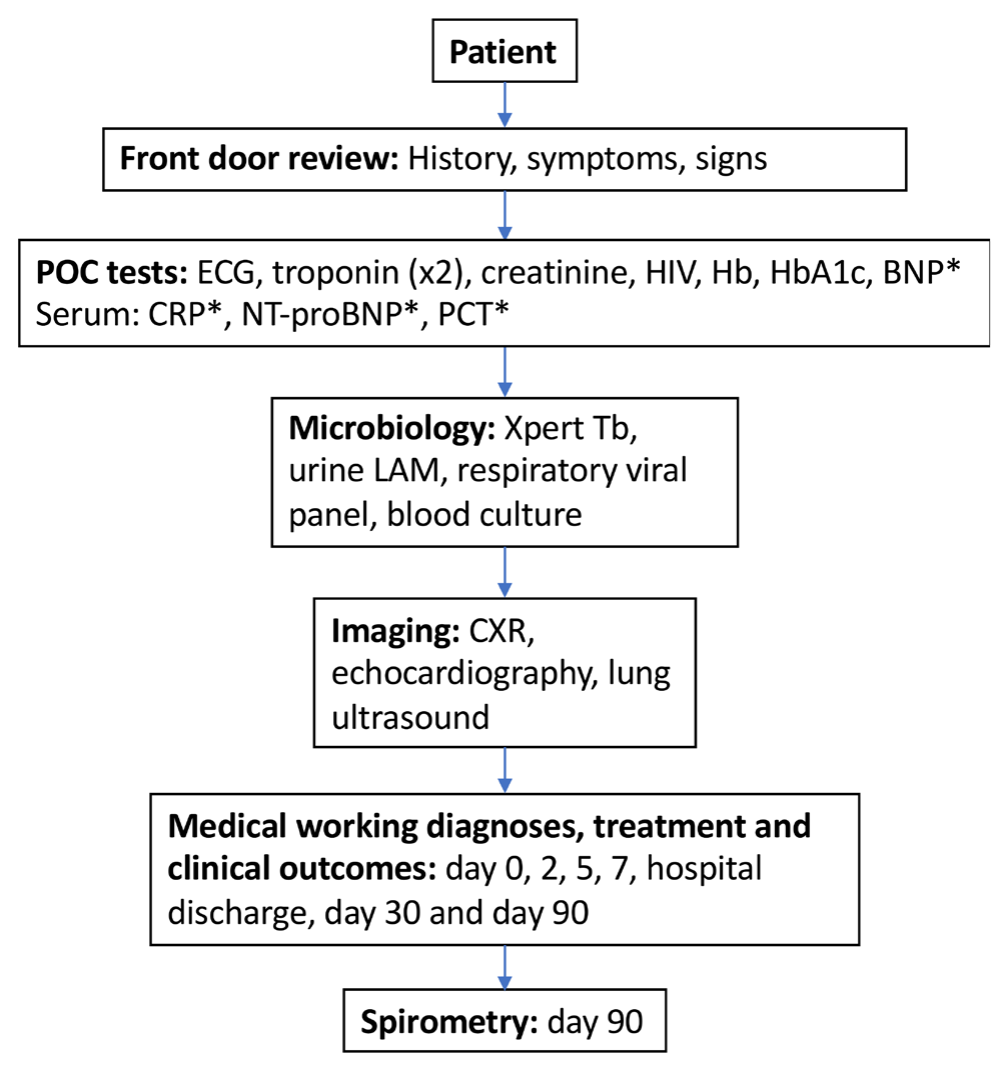
Participant flow through study investigations. BNP: brain natriuretic peptide; CXR: chest x-ray; CRP: C-reactive protein; Hb: haemoglobin; HbA1c: glycated haemoglobin; HIV: human immunodeficiency virus; NT-proBNP: N-terminal pro-brain natriuretic peptide; PCT: procalcitonin; Tb: tuberculosis; urine LAM: urine lipoarabinomannan. *Index tests

We will provide enhanced diagnostic testing to determine estimates of disease pathology using gold standard definitions. Details on the tests that will be introduced by the study and those currently available in the health systems are shown in Table 2. Information on diagnostic test methodology are presented in supplementary file 1 as *Extended data* ^22^, where information on sample acquisition, laboratory processes, interpretation, quality assurance [Qa] and quality control [Qc]) can be found for each test ( Figure 1). Clinically relevant findings obtained through the research study will be recorded in the patients’ medical notes and will be made available to treating clinicians. All equipment used will be CE marked (conformation with European Union safety, health and environmental legislation).

### Diagnostic criteria and case definitions

Diagnostic criteria and case definitions are presented in Table 3. We conducted a systematic literature search to identify up-to-date case definitions that are practical to the resource-constrained context based on available parameters (see Box 1). Definitions may be updated in the event that more recent reference standards are published and accepted by national or international committees.

**Box 1. Selection of case definitions**

We conducted a systematic literature search to identify and examine case definitions and diagnostic methodology for conditions that lead to breathlessness (see supplementary file 2 in *Extended data* ^22^ for details). First, a literature search was conducted on MEDLINE in February 2024 and identified 2069 articles, to identify contemporary guidelines published since 2010. Second, grey literature was identified through national and international societies (list provided in supplementary file 2 ^22^). Third, articles were identified from the reference lists of screened studies and from author experience. Following title and abstract screening, we identified 101 articles for full review, of which 69 were included in the protocol development. Articles were considered if they included diagnostic guidance on the conditions listed in Table 3 (including disease sub-classification). Case definitions were reviewed on their practicality to the resource-constrained context and available parameters with a preference for case definitions that do not require multimodal imaging, specialist laboratory equipment or technical expertise that are not routinely available in the health system (see Figure 1). The decision of case definitions to take forward into the study was based on consensus of authors with subjects-specific expertise. For example, cardiac imaging experts (DM, DA, DO, SS) reviewed and agreed on cardiac-related case definitions; respiratory experts (SG, JR, BM, SS) reviewed and agreed on respiratory-related case definitions.

In instances of missing or incomplete data, we will follow methodologic guidance from the NIHR Health Technology Assessment Programme ^13^. Specifically we will: 1) identify suitable alternative reference standards; 2) agree on diagnoses through consensus; 3) use multiple imputation methods. For exploratory sensitivity analyses we will also use latent class methods to evaluate the performance of index tests ^51^.

Where required for consensus agreement, a panel consisting of at least three medical doctors with context-relevant experience will review all the cases to assign diagnoses. This will consist of a review of all information shown in Figure 1. Panellists will be blinded to results of index tests (brain-natriuretic peptide [BNP], procalcitonin [PCT] and C-reactive protein [CRP]). Each member will review the documents independently. In cases of discrepancies, consensus will be reached through discussion.

### Study size

**Figure 2.**
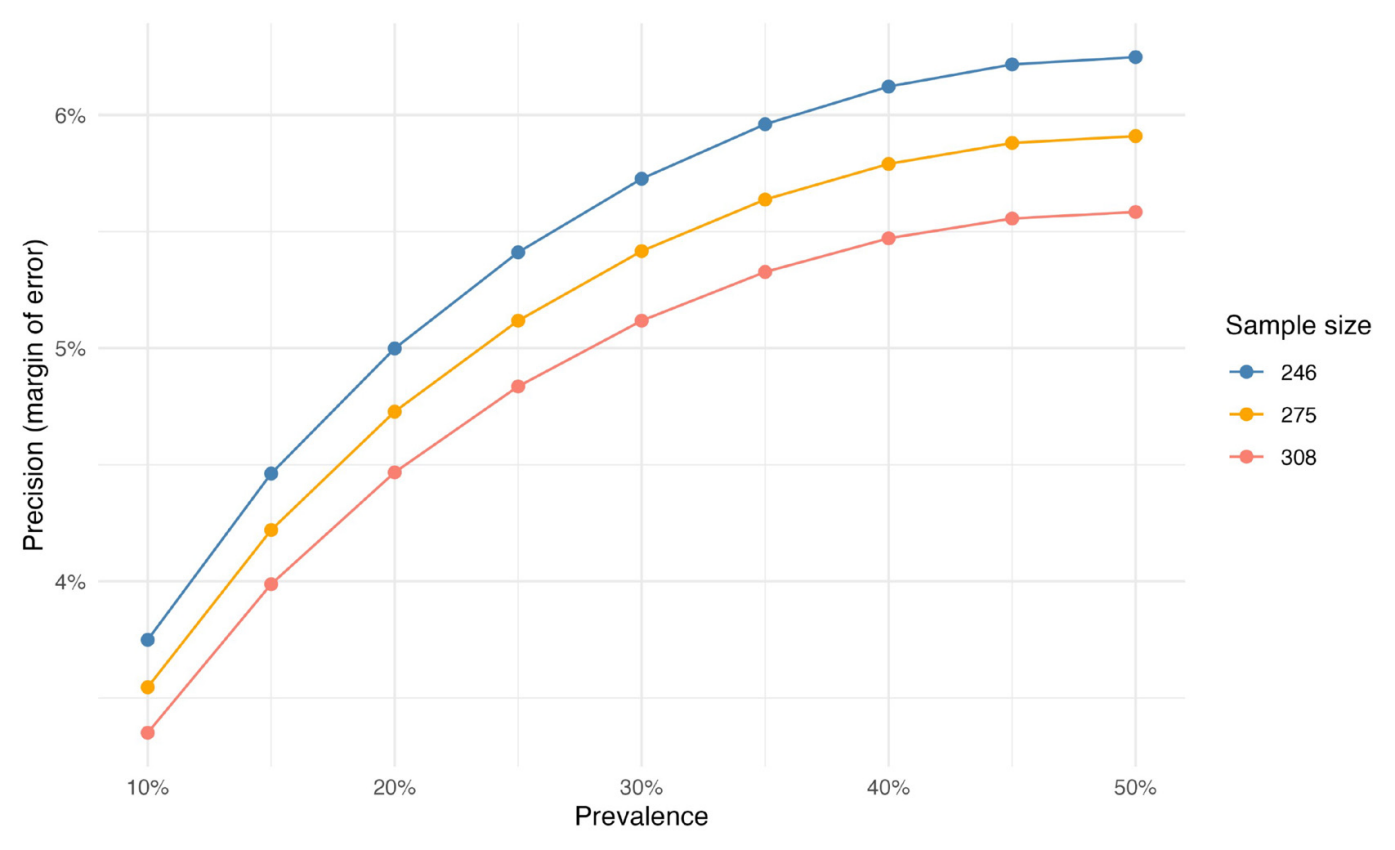
Sample size impact on precision of prevalence estimate.

We will aim to calculate prevalence to 5% precision (margin of error). For a given sample size, as prevalence estimates tend towards 50% the margin of error will be wider ( Figure 2). We have therefore powered this study on heart failure and pneumonia as these are likely to be the two most common presentations with expected prevalence of approximately 20% (estimates triangulated from local disease estimates and previous reports) ^1, 52^. Under these assumptions, at 5% precision, the sample size is 246. The target of 308 participants (20% total attrition) aims to offset losses from uninterpretable clinical images; clinical sample errors; and patient drop out. Figure 2 shows the precision in prevalence that we expect to achieve and how this will be affected by sample size.

### Analysis

The observational cohort study will be conducted and reported in line with STROBE guidelines ^53^; the diagnostic accuracy study will be reported in line with STARD 2015 guidelines ^54^; the prediction model will be reported in line with TRIPOD guidelines ^55^.

***Observational study*.** Descriptive statistics will be disaggregated by sex. Continuous variables without substantial skew and without outlying observations will be summarised by mean and standard deviation; continuous data with skew or outlying observations will be summarised by median and interquartile range. Prevalences will be reported alongside exact 95% binomial confidence intervals. Patient outcomes will include: in-hospital mortality, 30-day mortality, 90-day mortality, and an aggregate of readmission-free survival by day 90. Univariate and multivariate logistic regression analyses will be conducted to evaluate the impact of patient variables (such as diagnosis) on patient outcomes. In instances of missing data we will use multiple imputations using chained equations (MICE) where appropriate.

We will examine intra-observer and inter-observer reliability through calculation of intra-class correlation coefficients, coefficient of variation and Bland-Altman plots for continuous variables; concordance proportions and kappa statistics for categorical variables.

***Diagnostic accuracy study*.** We will compare the diagnostic accuracy of index tests with diagnoses (presence or absence of disease) made by established gold standard reference tests. To prevent information or incorporation bias, investigators will be blinded when interpreting all tests (i.e. blinded to the results of the index test when interpreting reference tests). Given that the significance of different performance measures varies depending on the setting, context, and use case, will report diagnostic performance using sensitivity, specificity, positive and negative predictive values, positive and negative likelihood ratios, diagnostic odds ratio, and the area under the receiver operating curve (AUROC), along with corresponding 95% confidence intervals. We will compare the performance index tests to reference standards (see Table 3). For exploratory sensitivity analyses we may also examine index test performance with latent variable models ^51^.

***Clinical prediction study*.** We will also explore whether a clinical prediction model can be developed to accurately diagnose and differentiate between common causes of breathlessness, using context appropriate clinical and biochemical markers (for example for the diagnosis of heart failure and differentiation from respiratory infections such as pneumonia). We will derive a multivariable prediction model; evaluate performance (with measures of discrimination [e.g. c-statistic] and calibration [e.g. calibration plots]); and internally validate the model (using bootstrap resampling methods). We will follow methodological guidance to conduct and report the study ^55, 56^, and will provide the model details to allow for external use and validation. This has the potential to inform the development of parsimonious clinical diagnostic algorithms.

***Economic analysis*.** We will present estimates of the units costs for each of the tests ( Figure 1). Each test will be costed according to a micro-costing approach. For POC tests we will estimate equipment and consumable costs per test and healthcare worker time costs using estimates of time to administer the test and obtain the results using healthcare worker salary costs for Malawi Ministry of Health funded staff costs. Clinical imaging costs will be based on equipment costs, maintenance, healthcare workers and an estimate of realistic number of tests conducted per year (e.g. at Queen Elizabeth Central Hospital). For microbiology, costing will be based on costs per test as charged by MLW laboratory.

## Study status

This study is currently in the follow-up phase.

## Ethical considerations

Ethics was obtained from LSTM (21-086; approved on 10.05.2022) and College of Medicine Research and Ethics Committee (COMREC), Malawi (P.11/21/3462; approved on 15.10.2021). Our reflexivity statement (see supplementary file 3 in *Extended data* ^22^) describes how we have promoted equity in our international research partnership ^57^.

Liverpool School of Tropical Medicine (LSTM) provided sponsorship for this work (as a nested component within the MultiLink study) ^12^.

## Discussion

There are few published epidemiological studies that investigate breathlessness in adult patients who present to hospital in Africa, particularly in the lowest income countries ^5, 6^.

Our recent systematic review of chronic disease data from sub-Saharan African hospitals revealed inconsistent case definitions, limited use of diagnostic tools, and a significant gap in adopting gold standard diagnostic approaches ^11^. For instance, less than one-third of heart failure studies employed echocardiography, the recommended imaging for diagnosis confirmation, and none of the studies reported myocardial infarction or COPD according to gold standard criteria. Case definitions in the field of breathlessness diagnoses are frequently inconsistent. We have carefully considered and developed thorough, transparent and relevant case definitions that are applicable to the resource-constrained context based on available parameters, and informed by review of existing guidelines.

There are a number of methodological considerations we have used to improve the strength of this study. Our eligibility criteria for breathless patients is inclusive of both symptoms and physiological parameters which will allow us to quantify the proportion of dyspnoeic patients with or without abnormal physiology. Enhanced testing will be provided alongside quality assurance and quality control for each diagnostic modality to ensure we obtain accurate results (see supplementary file 1 for details ^22^). Investigators will be blinded to avoid information or incorporation bias in the diagnostic accuracy study. We will utilise lung ultrasound, echocardiography and chest x-ray in recognition that the combined use of these tests enhances diagnostic accuracy for heart failure and pneumonia diagnoses ^58– 60^. Recruitment from a central and a district government hospital will provide valuable information from different levels of the Malawi health system to help inform the development of cost-effective diagnostic and treatment algorithms. 

This study has limitations we aimed to mitigate. First, due to limited resources, certain diagnoses such as lung fibrosis and bronchiectasis require high-resolution CT scanning, which is not available. However, in this study we focus on: 1) high burden conditions; 2) with risk of deterioration if not promptly treated; 3) that are treatable in the district hospital setting. Second, spirometry for COPD diagnosis will occur at day 90 (to allow for recovery from acute illness), with inherent susceptibility to survivorship bias. However, previous research suggests COPD diagnoses are uncommon in sub-Saharan hospital admissions ^11^. Furthermore, in instances of missing data required to categorise disease outcomes, we will follow methodological guidelines ^13^. Third, we will exclude pregnant women from this study as other research programmes specifically examine these populations in Malawi. Fourth, the majority of diagnostic guidelines identified originate from high income settings. We have prioritised internationally accepted criteria for inclusion in this study; however, future work is needed for local guideline development.

To date, little attention has been given to a thorough assessment of the underlying causes of breathlessness, a common hospital presentation in sub-Saharan Africa. Our objective is to produce a comprehensive, useful, and accessible description of the causes of breathlessness (dyspnoea and hypoxemia) in this region. Through our nested diagnostic studies, we aim to develop parsimonious, context-appropriate diagnostic algorithms. While practical diagnostic algorithms have been proposed for high-income settings (e.g. for heart failure diagnosis) ^61, 62^, our study aims develop algorithms suitable for low-resource settings. This will likely involve integration of readily available clinical information, point-of-care biomarkers, and imaging (e.g. point-of-care ultrasound) ^61– 64^. We will apply health systems frameworks ^65^, and conduct a diagnostic cost analysis to offer recommendations for prioritisation and implementation strategies within healthcare systems.

This study aligns with the 76 ^th^ World Health Assembly (WHA) resolution on *‘Strengthening diagnostics capacity’* ^3^, and the 76 ^th^ WHA resolution on *‘Integrated emergency, critical and operative care for universal health coverage and protection from health emergencies’* ^66^. It also ties in with calls from the 2021 Lancet commissions on diagnostics ^4^, and Global Acute Care Advocacy group ^67^. Our goal is to enhance diagnostic capabilities in acute care systems and to improve quality of comprehensive clinical care (and therefore outcomes) for acutely ill people in low-resource settings.

## Ethics and consent

Ethics was obtained from LSTM (21-086; approved on 10.05.2022) and College of Medicine Research and Ethics Committee (COMREC), Malawi (P.11/21/3462; approved on 15.10.2021).

Written consent will be obtained from all participants if they have capacity. If they do not have capacity, written personal or professional consultee (proxy) assent will be obtained in line with ethical approval. In instances where the participant subsequently regains capacity, we will we will obtain retrospective consent. Full details of the consent process has been published in the MultiLink manuscript ^12^.

## Data Availability

No data are associated with this article. Open Science Framework: Causes, outcomes and diagnosis of acute breathlessness hospital admissions in Malawi: a protocol for a multicentre prospective cohort study. https://doi.org/10.17605/OSF.IO/ZT9FD ^22^. This project contains the following extended data: Supplementary file 1: methods appendix Supplementary file 2: literature search appendix Supplementary file 3: reflexivity statement Supplementary file 4: STROBE checklist Data are available under the terms of the Creative Commons Zero "No rights reserved" data waiver (CC0 1.0 Public domain dedication). **Malawi-Liverpool-Wellcome Trust Clinical Research Programme, Blantyre, Malawi** Brigitte Denis, George Selemani, Joshua Kaphika, Catherine Anscombe, Joseph Bwanali, Linda Gondwe, Maureen Kandiero, Mirriam Machonjo, Kate Mangulama, Mavis Menyere, Alice Mnyanga, Alfred Muyaya, Doris Shani **Johns Hopkins Research Project Laboratory, Blantyre, Malawi** Lameck Manda, Enock Jumbe **Achikondi Women’s Health Clinic, Lilongwe, Malawi** Charity Salima **Kilimanjaro Clinical Research Institute, Moshi, Tanzania** Sarah Urasa **Liverpool School of Tropical Medicine, Liverpool, UK** Laura Rosu, Amy Smith, Sarah White **University of Liverpool, Liverpool, UK** Elly Wallis **Liverpool John Moores University, Liverpool, UK** Luca Howard
