# Supplementary material for: Causes, outcomes and diagnosis of acute breathlessness hospital admissions in Malawi: protocol for a multicentre prospective cohort study
Source: Wellcome Open Res. 2024 Apr 17;9:205. [Version 1] doi: 10.12688/wellcomeopenres.21041.1 (PMC11327656; doi:10.12688/wellcomeopenres.21041.1)
Supplement: Peer review history

# Reviewer report: Reviewer response for version 1

**DOI:** 10.21956/wellcomeopenres.23277.r85784

**Published:** 2024-08-15

**Authors:** Rudolf K F Oliveira, Thais F C Menezes

**Affiliations:** 1 Division of Respiratory Diseases, Federal University of São Paulo, São Paulo, Sao Paulo, Brazil; 2 Division of Respiratory Diseases, Federal University of Sao Paulo, Sao Paulo, Sao Paulo, Brazil

Acute breathlessness is a common complaint in the emergency department. Also known as dyspnea, it can be secondary to various diseases, some with high morbidity and mortality. Therefore, accurately diagnosing acute breathlessness and quickly referring the patient for appropriate treatment is crucial, yet challenging. This challenge becomes even more significant in low-income countries, where access to rapid and efficient diagnostic methods is often limited.

 Despite the high prevalence of acute dyspnea among the causes of hospitalization, a detailed understanding of the causes and outcomes of this condition in hospitalized adults remains insufficient, particularly in settings like Malawi, in sub-Saharan Africa. The study "Causes, outcomes and diagnosis of acute breathlessness hospital admissions in Malawi: protocol for a multicenter prospective cohort study" by Stephen A. Spencer et al. emerges as a pioneering initiative aimed at filling this critical knowledge gap.

 This multicenter prospective cohort study seeks to delineate the underlying causes of breathlessness in adult patients admitted to hospitals in Malawi and report disease prevalence. It also aims to assess clinical outcomes (mortality and hospital readmission 90 days after discharge) and the accuracy of diagnostic biomarkers to differentiate two majors causes of dyspnea in the literature: heart failure vs respiratory infections. The evaluation of patients from two distinct hospital centers (Queen Elizabeth Central Hospital and Chiradzulu District Hospital) enhances the study, as it allows  capturing a broad diversity of clinical scenarios, from an urban tertiary referral hospital to a secondary rural district hospital, reflecting the variety of challenges faced by resource-limited health systems.

 The importance of this study cannot be overstated. In low-income countries, where healthcare infrastructure is often fragile, the lack of accurate diagnostics can result in inadequate or delayed treatments, leading to severe and often fatal consequences for patients. By investigating the efficacy of biomarkers such as brain natriuretic peptides and C-reactive protein, the study offers valuable insights into how diagnostic processes can be optimized with the available resources.

 The study's methodological approach is both notable and robust. The prospective design and the inclusion of model calibration and discrimination analyses suggest that the results could be both reliable and applicable to similar settings. However, the reliance on data from only two hospital centers should be considered, as it may limit the generalizability of the findings. It would be important to acknowledge this limitation and suggest external validation in different geographic and demographic contexts. Although the use of bootstrap resampling has its critics, it remains a suitable methodological choice for addressing uncertainty in smaller samples, offering more realistic confidence intervals and robust internal validation. Nevertheless, advocating for future external validation to confirm the model's generalizability is essential. Furthermore, when interpreting statistical measures such as c-statistic and calibration plots, the study should not only present these metrics but also provide a thorough discussion on how well the model's predictions align with observed event rates, particularly across relevant clinical subgroups.

 A notable limitation of this study is the reliance on diagnostic methods that do not always meet the gold standard, primarily due to the unavailability of such tests in the regions where the research is conducted. In many low-income countries, financial and social constraints limit access to more accurate, albeit costly, diagnostic tools. Consequently, the study must depend on alternative methods, which could result in less precise diagnoses and potentially impact the validity of the findings. To address this limitation, the study incorporates enhanced diagnostic testing where feasible, supported by rigorous quality assurance and control processes for each diagnostic tool used. In instances of missing data, the researchers will follow established methodological guidelines, employing recommended statistical methods to handle the missing information. These procedures aim to minimize bias and ensure the reliability of the study’s conclusions, maintaining the robustness and accuracy of the analysis even when data is incomplete.

 In conclusion, the study protocol outlined by Spencer et al. is a valuable contribution to medical literature and its results could impact the diagnostic and therapeutic approaches for patients with acute breathlessness in resource-limited settings. It is hoped that this study will not only improve the understanding of acute breathlessness in Malawi but also offer a replicable model for other regions facing similar healthcare challenges. This is a vital step towards improving global health and reducing disparities in access to quality care.

Is the study design appropriate for the research question?

Yes

Is the rationale for, and objectives of, the study clearly described?

Yes

Are sufficient details of the methods provided to allow replication by others?

Yes

Are the datasets clearly presented in a useable and accessible format?

Not applicable

Reviewer Expertise:

Respiratory Medicine, Exercise Physiology, Pulmonary Circulation

We confirm that we have read this submission and believe that we have an appropriate level of expertise to confirm that it is of an acceptable scientific standard.

# Reviewer report: Reviewer response for version 1

**DOI:** 10.21956/wellcomeopenres.23277.r82703

**Published:** 2024-06-05

**Authors:** C. Louise Thwaites

**Affiliations:** 1 Oxford University Clinical Research Unit, Ho Chi Minh City, Vietnam

This is a protocol for a study that is already underway (in the follow up phase). It addresses an important unmet need and shows a logical and appropriate approach to the research question. The data provided will be of great value in understanding the causes of breathlessness in this setting.

Is the study design appropriate for the research question?

Yes

Is the rationale for, and objectives of, the study clearly described?

Yes

Are sufficient details of the methods provided to allow replication by others?

Yes

Are the datasets clearly presented in a useable and accessible format?

Not applicable

Reviewer Expertise:

Critical care

I confirm that I have read this submission and believe that I have an appropriate level of expertise to confirm that it is of an acceptable scientific standard.
